# Kids Safe and Smokefree (KiSS): a randomized controlled trial of a multilevel intervention to reduce secondhand tobacco smoke exposure in children

**DOI:** 10.1186/1471-2458-13-792

**Published:** 2013-08-30

**Authors:** Stephen J Lepore, Jonathan P Winickoff, Beth Moughan, Tyra C Bryant-Stephens, Daniel R Taylor, David Fleece, Adam Davey, Uma S Nair, Melissa Godfrey, Bradley N Collins

**Affiliations:** 1 Department of Public Health, Temple University, 1301 Cecil B. Moore Ave, Ritter Annex, 9th Floor, Philadelphia, PA, 19122, USA; 2 Department of Pediatrics, Massachusetts General Hospital for Children, Boston, USA; 3 Department of Pediatrics, Temple University School of Medicine, Philadelphia, USA; 4 Department of General Pediatrics, Children’s Hospital of Philadelphia, Philadelphia, USA; 5 Department of Pediatrics, St. Christopher’s Hospital for Children, Philadelphia, USA

**Keywords:** Secondhand smoke, Pediatrics, Randomized controlled trial, Prevention, Tobacco control, Smoking cessation, Health services, Electronic health records

## Abstract

**Background:**

Secondhand smoke exposure (SHSe) harms children’s health, yet effective interventions to reduce child SHSe in the home and car have proven difficult to operationalize in pediatric practice. A multilevel intervention combining pediatric healthcare providers’ advice with behavioral counseling and navigation to pharmacological cessation aids may improve SHSe control in pediatric populations.

**Methods/design:**

This trial uses a randomized, two-group design with three measurement periods: pre-intervention, end of treatment and 12-month follow-up. Smoking parents of children < 11-years-old are recruited from pediatric clinics. The clinic-level intervention includes integrating tobacco intervention guideline prompts into electronic health record screens. The prompts guide providers to ask all parents about child SHSe, advise about SHSe harms, and refer smokers to cessation resources. After receiving clinic intervention, eligible parents are randomized to receive: (a) a 3-month telephone-based behavioral counseling intervention designed to promote reduction in child SHSe, parent smoking cessation, and navigation to access nicotine replacement therapy or cessation medication or (b) an attention control nutrition education intervention. Healthcare providers and assessors are blind to group assignment. Cotinine is used to bioverify child SHSe (primary outcome) and parent quit status.

**Discussion:**

This study tests an innovative multilevel approach to reducing child SHSe. The approach is sustainable, because clinics can easily integrate the tobacco intervention prompts related to “ask, advise, and refer” guidelines into electronic health records and refer smokers to free evidence-based behavioral counseling interventions, such as state quitlines.

**Trial registration:**

NCT01745393 (clinicaltrials.gov).

## Background

Child secondhand smoke exposure (SHSe) is linked to asthma, respiratory illness, otitis, hospitalization rates, headaches, sudden infant death, and behavior problems [1-4]. Because parental smoking is a primary source of SHSe in children, the American Academy of Pediatrics developed pediatric tobacco-intervention guidelines [5] that address parental smoking. Pediatric healthcare visits provide a teachable moment to increase parents’ awareness of the adverse effects of SHSe on children and motivate them to protect children from SHSe [6]. However, there are barriers to effective pediatric-clinic interventions. For example, competing demands, time limitations, and systems barriers may prohibit offering medications to address parents’ nicotine addiction or providing in-depth counseling and training in self-regulatory skills that can alter smoking behavior [7,8]. The Kids Safe and Smokefree (KiSS) program addresses these limitations via a multilevel intervention model that integrates a pediatric clinic-level intervention with more intensive individual-level behavioral counseling and navigation to community-level services for nicotine dependence.

The KiSS clinic-level intervention focuses on improving the quality of clinic-delivered tobacco-related messages for parents. It emphasizes three elements, known as “Ask, Advise, and Refer” (AAR): Ask about child SHSe, Advise about the harms of SHSe and benefits of reducing SHSe, and Refer to cessation resources. The clinic AAR intervention can motivate and assist parents to take the initial actions to protect their children from SHSe. The KiSS behavioral health intervention then provides more intensive intervention that may be necessary to promote smoking behavior change. The behavioral intervention combines personalized, intensive family-centered counseling, smoking urge management and coping skills training and social support with community-level systems navigation to facilitate access to and effective use of no-cost nicotine replacement therapy and reimbursable cessation medication. The behavioral intervention includes a home visit to introduce intervention concepts and initiate skills training around reducing children’s SHSe in the home and car as a primary step toward preparing to quit smoking. The home is a critical target of intervention because restrictions on smoking across the U.S. do not typically extend to private homes and cars - contexts in which child SHSe is greatest. Weekly telephone counseling following the home visit initially emphasizes reducing child SHSe and then progresses to address smoking cessation and relapse prevention.

The design and sequencing of procedures in our trial were influenced in part by a pivotal review of smoking cessation interventions in medical practice. In that review, Kottke [9] concluded that information to a smoker from one type of personnel (e.g., clinician) may potentiate information from another type of personnel (e.g., health counselor), and the number and duration of reinforcing sessions are related to cessation success. Kottke’s review focused on cessation, but we believe the same effects of multiple message sources and repeated “doses” of advice can be harnessed for SHSe reduction using an integrated multilevel, multimodal intervention. We will evaluate efficacy of the intervention in predominantly low-income, urban and minority communities with excess SHSe-related morbidity and mortality risk.

To date, most pediatric SHSe interventions adopt what Anderson [10] has described as a single level of analysis--focusing either on environmental factors (e.g., smoke-free policies), social factors (e.g., pediatrician recommendation), or individual factors (e.g., motivation to change). The failure to develop a multilevel approach has impeded progress in the field because it is well established that smoking is multidetermined, and when an intervention targets a single level of a multidetermined behavior, that intervention provides insufficient elements to maintain healthy behavior change. For example, interventions that target one particular cause (e.g., limited knowledge about harm) might not address other relevant causes (e.g., nicotine addiction). Hence, a multilevel approach is likely to be more effective than a single-level approach.

The multilevel KiSS model follows recent recommendations to advance the science of health behavior change by testing a multilevel approach that addresses individual, group, and environmental influences simultaneously and over time. The model minimizes burden on clinicians, but the clinician still acts as a credible gateway to the more intensive intervention for smoking parents. The specific components of the clinic- and individual-level interventions are informed by the literature, including our preliminary studies and theory [11-13]. For example, research links social support [14], coping skills [15], and self-efficacy [16] to smoking behavior change. The KiSS interventions have elements shown to be associated with improved social support [17], coping skills [18], and self-efficacy [19]. Other non-program factors, such as psychological symptoms [20], nicotine dependence [21], and presence of other smokers in home [22], are known predictors of smoking outcomes and might moderate intervention efficacy.

### Aims and hypotheses

Aim 1: Test the primary hypothesis that an intervention integrating pediatric clinic-level quality improvement with individual-level behavioral counseling (AAR + BC) will be more effective in reducing children’s SHSe than a clinic-level quality improvement plus attention control intervention (AAR + AC). *Hypothesis:* Compared with children in the AAR + AC condition, those in the AAR + BC condition will have significantly greater reductions in SHSe from baseline to 3- and 12-month follow-up.

Aim 2: Test the secondary hypothesis that AAR + BC will be more effective in increasing parental quit rates than AAR + AC. *Hypothesis:* Compared with parents in the AAR + AC condition, parents in the AAR + BC condition will have a significantly greater smoking abstinence at 3- and 12-month follow-up.

Aim 3: Test hypotheses that social cognitive variables (social support, urge management coping skills, self-efficacy) will mediate effects of the AAR + BC intervention on outcomes. *Hypothesis*: Compared with parents in the AAR + AC condition, parents in the AAR + BC condition will report greater social support, coping skills, and self-efficacy related to smoking cessation and SHSe reduction from baseline to 3- and 12-month follow-up. In turn, these changes will account for between-group differences in child SHSe and parent cessation outcomes.

Aim 4: Explore factors that may affect outcomes and moderate intervention effects, including presence of other smokers at home, level of nicotine dependence, and depressive symptoms.

## Methods/design

This study uses a randomized, two-group (experimental vs. attention control) design with three measurement periods: pre-intervention, end of treatment (3 months) and long-term follow-up (12 months). Smoking parents are recruited through urban pediatric healthcare clinics. The primary outcome of interest is child SHSe and the secondary outcome of interest is parent smoking abstinence. The study design is guided by CONSORT criteria [23] and is approved by the relevant Institutional Review Boards (Temple University protocol number 20045). In the clinics, providers will ask parents about child SHSe, provide information on the harms of child SHSe and on the benefits of reducing child SHSe, and refer parents to cessation resources that include the KiSS program. Eligible parents are randomized either to an individual behavioral telephone counseling intervention that focuses on reducing child SHSe and parent smoking cessation or (b) an individual telephone health education attention control intervention that focuses on improving family nutrition on a budget.

### Participants

**Figure 1:**
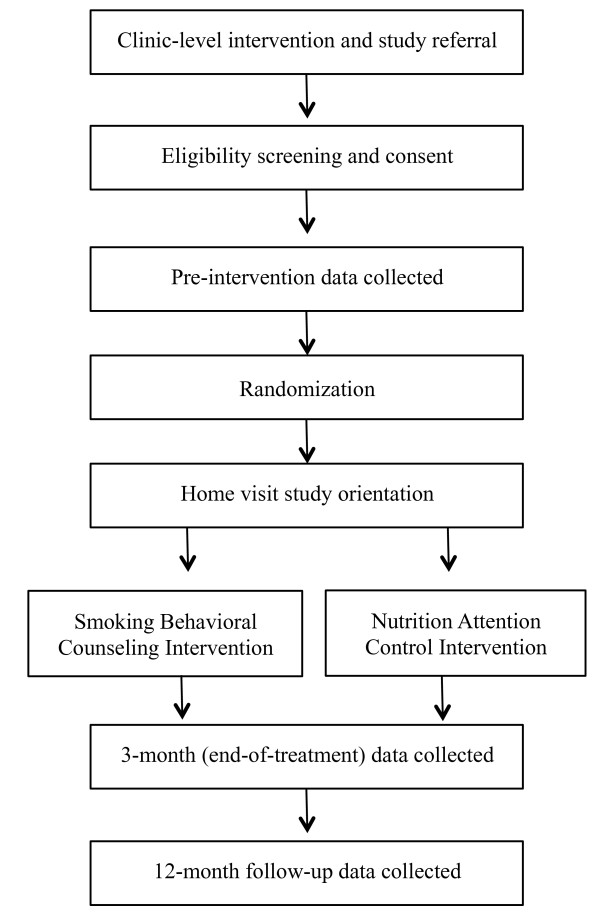
Flowchart of recruitment, intervention and assessment for Kids Safe & Smokefree (KiSS) trial.

The pediatric clinics from which the sample will be drawn service predominantly low-income, racial- and ethnic-minority families. Eligibility inclusion criteria for parents are: Received pediatric clinic-level intervention, English-speaking, aged 18 years or older, report daily smoking, report that a child in the home under the age of 11-years old is exposed daily to cigarettes in the home/car. Exclusion criteria include patient-reported psychiatric disturbance, pregnancy, or consumption of three or more alcoholic beverages per day. Figure 1 shows the participant flow through clinic intervention, enrollment, intervention, and data collection.

### Procedures

Pediatric healthcare providers in partnering clinics will be trained to provide brief advice about child SHSe and to refer assenting parents to the KiSS research staff. The sampling frame includes any parent who meets the eligibility requirements following receipt of the clinic intervention and referral. Trained research assistants consent eligible participants and collect self-report data at baseline, 3-month end-of-treatment, and 12-month follow-up via structured telephone interviews. Interviewers are blind to participants’ intervention condition. Various procedures are used to reduce attrition, including reminder postcards and text messages, flexible scheduling, and financial retention incentives for completing different phases of the study.

Randomization is stratified by site and race using randomized permuted blocks of varying lengths. Because site and strata were not fixed design characteristics, the maximum number of allocations was generated within each combination of site and stratum. The project biostatistician provided the allocations to the data collection team in opaque sealed security envelopes. Randomization was seeded using values obtained from random.org. Participants are randomized prior to an orientation home visit that follows the baseline interview. Once a home visit appointment is confirmed, a research assistant takes the top envelope from the allocation pile to bring to the home visit. The envelope is opened immediately prior to the home visit to reveal the condition.

During the home visit, participants receive a binder with materials that are relevant to their assigned intervention condition and they provide a sample of the child’s urine for baseline cotinine assessment. The child’s urine is collected again at the 12-month follow-up. Parents who report quitting smoking at 12-month follow-up are asked to provide a biospecimen for cotinine verification of quit status.

#### Interventions

As part of the KiSS project, participating clinics have modified their electronic health records (EHR) to include prompts to healthcare providers to address parental smoking and to record information on SHSe in the EHR. The KiSS PIs coordinate pediatric providers’ training and ongoing AAR monitoring in collaboration with clinic liaison co-investigators. During a clinic visit, any parent accompanying the child who is a smoker is referred to KiSS. Thus, all study participants receive clinic-level intervention related to child SHSe, as recommended by the American Academy of Pediatrics, prior to randomization. Once randomized, participants will receive either additional individual-level intervention related to their smoking and child SHSe or education about nutrition on a budget, depending on their assignment.

##### Clinic intervention using AAR (both groups)

The clinic-level component of KiSS was modeled on the guiding principles underlying the standalone Clinical Effort Against Secondhand Smoke Exposure (CEASE) program for addressing parental smoking in pediatric clinics [24,25]. However, unlike CEASE, the KiSS AAR intervention is integrated within each clinic’s EHR system. Key goals of the KiSS AAR intervention are to (1) identify parents who smoke in the child's home, (2) counsel smokers about tobacco use and establishing no smoking policies around children; (3) provide smoking intervention and medication referral resources to the parent (including referral to the KiSS project and NRT prescription); (4) record information in the EHR to facilitate ongoing follow-up regarding the child’s SHSe. During a pediatric clinic visit, the healthcare provider is prompted to ask the parent about the child’s exposure to SHS, enter the diagnosis into the health record, and advise the parent about the potential harms of child SHSe and benefits of reducing exposure. Finally, the provider gives parents a brochure, *Secondhand Tobacco Smoke and the Health of Your Family* (EPA 402/F/09-044) from the Environmental Protection Agency, a printout of local and state resources for people who are “Ready to Quit” that includes information about cessation medication and nicotine replacement products, and inform parents about the KiSS program and refers assenting parent to KiSS staff (e.g., via EHR auto-fax). There are two primary differences between the KiSS AAR and CEASE: KiSS works within the EHR rather than using any paper-based systems to prompt AAR steps and shifts the burden of advice, counseling and navigation to nicotine replacement therapy and medication from the pediatric provider to a behavioral health counselor.

Integrating KiSS AAR into routine clinic operations involves adoption, implementation, and maintenance phases. During adoption, the clinic liaisons meet with the investigators to design and implement the AAR interface in clinics’ EHR system for identifying children exposed to SHS and referring interested parents to KiSS. The program is then implemented in each participating clinic using an academic detailing approach, including talking with providers about the program, promoting AAR steps to improve the quality of patient care, installing KiSS program posters and placing brochures in waiting and clinic exam rooms. In the maintenance phase, clinic liaisons receive quarterly reports on clinic performance that includes number of referrals received and information on whether referred parents report receiving oral and written materials on the harms of child SHSe, the benefits of reducing SHS, and local smoking cessation support services.

##### Smoking intervention: behavioral counseling (AAR + BC)

Trained health counselors deliver the BC intervention. Training consists of role-playing, didactic sessions, and readings on nicotine dependence and intervention. Counselors learn about the provision of standard cognitive-behavioral counseling strategies to facilitate smoking behavior change, how to help participants to set short-term treatment goals, how to provide social support and positive reinforcement, how to facilitate problem solving to overcome behavior change barriers, and how to provide guidance and advice about existing services and coverage to obtain nicotine replacement products. Counselors also receive weekly supervision to review specific cases and adherence to treatment protocols. The telephone counseling sessions are audiotaped and a subset of the sessions are reviewed to ensure adherence to the manualized treatment protocol.

Prior to beginning telephone counseling related to smoking and child SHSe, KiSS staff members visit participants in their home to provide an orientation to the intervention program, share a program binder that is used in conjunction with the telephone counseling, and collect a baseline urine sample from the target child for cotinine assessment. The BC intervention consists of five telephone-counseling sessions, interspersed with “check-in” retention calls, delivered over 12 weeks. The BC intervention explicitly extends pediatric provider advice parents received at a recent clinic visit. The BC intervention consists of evidence-based strategies to promote SHSe reduction, smoking cessation and relapse prevention, including health education and social support to guide child SHSe-reduction efforts, cognitive-behavioral counseling to promote coping skills acquisition for smoking urge and stress management, and motivational interviewing techniques to facilitate collaborative, personalized treatment. The intervention process is guided by ecological and associative learning theories (e.g., Social Cognitive Theory; Behavioral Ecological Model [12,26]) and uses components from the FRESH (Family Rules for Establishing Smoke-free Homes) [11,22] intervention. Therefore, counselors guide participants to break down smoking behavior change efforts into manageable steps in preparation for cessation by utilizing a behavioral shaping approach that reinforces progress on short-term goals to maintain motivation and confidence in achieving long-term smoking cessation outcomes. For example, smokers will first focus on child health and SHSe reduction (e.g., setting dates to establish home smoking bans) and use behavioral strategies to facilitate the short-term goal in preparation for quitting smoking and maintaining smoking abstinence.

The KiSS BC program is distinct from FRESH in two key dimensions. First, KiSS counselors review and reinforce the information conveyed by the pediatric healthcare provider—an intervention component that is absent from FRESH. Second, KiSS counselors navigate participants to additional smoking cessation services that are publically available. For example, counselors will encourage and provide logistical support that helps participants to access smoking quitlines and nicotine replacement therapy and prescription cessation medications. Counselors will educate participants about existing resources, how to access them, and problem-solve barriers to adherence to cessation medication.

##### Attention control: nutrition education (AAR + AC)

Trained health counselors deliver the AC intervention. Training, intervention orientation, and telephone counseling procedures mirror those of the BC arm, but focus on improving nutrition on a budget (e.g., learning about national guidelines related to fruit and vegetable consumption). The purpose of the AAR + AC group is to equate attention and contact between the two experimental conditions while providing distinctly different intervention content. Included in the home visit materials is a tool kit developed by Sesame Street Workshop, *Food for Thought: Eating Well on a Budget*[27]. The kit includes DVD videos on nutrition on a budget, nutrition guidelines, recipe cards, and colorful books, activity suggestions, and videos on fruit and vegetable consumption for children.

### Measures

#### Primary and secondary outcome variables

Participant self-report data is collected via telephone interviews. The primary dependent variable—child SHSe—will be measured in two ways. First, child cotinine will be collected in urine samples taken at baseline and the 12-month follow-up. Second, child SHSe will be measured by parental report of the number of cigarettes to which the child is exposed each day during the 7 days prior to all assessment periods [28]. The secondary dependent variable—parent’s smoking cessation—will be assessed via parent-reported 7-day point prevalence abstinence for the 7 days prior to assessment at 3- and 12-month follow-up. The 12-month parent cessation report will be cotinine-verified.

#### Covariates, mediators, and moderators

Three variables will be measured as potential covariates and possible moderators of intervention effects: nicotine dependence will be measured with the Fagerström Test for Nicotine Dependence [29], symptoms of depression will be measured with the Center for Epidemiological Studies 10-item depression scale [30], and a single self-report item will assess number of individuals who smoke daily in the home [31]. Additional background factors, including demographics and smoking history variables will be assessed for possible association with outcomes. Three variables will be measured as potential intervention mediators: program support for smoking cessation will be measured with the short form of the Partner/Significant Other Interaction Questionnaire [32] (adapted to focus on KiSS program support), coping skills will be measured with the Urge Management Coping Skills measure developed by the authors based on urge management coping strategies identified by O’Connell et al. [33], and DiClemente’s Self-Efficacy measure [34] will be used to measure people’s confidence in their ability to refrain from smoking in different situations.

#### Process measures and treatment fidelity

Intervention processes will be assessed in both conditions at multiple time points, with multiple methods, and at each level of intervention: AAR and BC. Process data will include counselors’ observations (e.g., number of sessions, participant engagement ratings) and participants’ reports (e.g., receipt of pediatrician advice and informational brochures). AAR treatment fidelity will be assessed at the clinic level and BC fidelity at the individual counselor level.

### Analytic plan

Child outcomes are continuous and the parent outcome is dichotomous. The predictor is intervention group (AAR + BC vs. AAR + AC). Additional covariates may include number of smokers in home, level of nicotine dependence, depressive symptoms, demographic, and smoking history. ANCOVA will be used for continuous outcomes and logistic regression will be used for the dichotomous outcome. Intention-to-treat analyses will be performed using multiple imputations with careful attention to missing data mechanisms. Potential covariates and effect modifiers will be considered as warranted. The influence of mediator variables, such as urge management coping skills, will be assessed using bootstrapping techniques that provide estimates of the indirect effects and test for their significance via confidence intervals [35]. The SPSS macro developed by Preacher and Hayes to test multiple mediation models will be used to carry out the mediation analysis [36].

Evidence for hypothesis 1 (child SHSe) will be provided by a significant negative coefficient for the indicator of AAR + BC participation. Evidence for hypothesis 2 (parent quit) will be provided by a significant positive coefficient for the indicator of AAR + BC participation. Evidence for hypothesis 3 (mediators) will be provided by significant associations between AAR + BC participation and social cognitive variables (positive) and between social cognitive variables and child SHSe (negative) and parent smoking (positive) outcomes. Evidence for hypothesis 4 (moderators) is exploratory; evidence for effect modification will be provided by significant coefficients associated with multiplicative composites between AAR + BC participation and potential moderating variables.

### Power analysis

Sample size was determined for the primary outcome (child cotinine) assuming power ≥ .80, α = .025, and a modest effect size (Cohen’s d = .20 - .30) [37]. Power estimates were derived from preliminary analysis of data from the FRESH trial [38], which indicated d ≥ .23 for child cotinine. Attrition is estimated to range from 25% to 30%. Based on these assumptions, sample size for recruitment was set at 166 per intervention arm (332 total).

## Discussion

Current approaches to pediatric SHSe reduction have limitations that weaken potential effectiveness when implemented at a single level. The KiSS intervention addresses these limitations through an integrated multilevel approach to addressing parental smoking. The end result of this project will be a novel, efficacious and translatable model for addressing the significant public health problem of child SHSe. Delivering the intervention in communities that have the highest prevalence of tobacco use and tobacco-related morbidity and mortality could produce the greatest public health benefits. Existing, publically-supported community-based providers of smoking cessation services, such as quitlines, and large urban pediatric clinics that serve high-risk populations could adopt this model by developing mutually advantageous partnerships: Clinics could provide referrals to established service providers who, in turn, could offer home-based behavioral counseling and systems navigation. Findings from the secondary mediator and moderator aims will inform science and theory in this field by identifying how and for whom the intervention works.

The significance of this multilevel approach could be realized through its influence on improved clinical practice by: a) improving tobacco intervention training and resources, b) simplifying and minimizing steps necessary for pediatric providers to adhere to tobacco intervention guidelines, and c) providing clinics with a parental smoking referral resource that helps parents get tobacco dependence treatment medications and addresses tobacco use and exposure in contexts where children’s SHSe is the greatest. If successful, this approach could inform science by providing evidence about the efficacy of integrating pediatrics and behavioral health approaches. While existing evidence-based practice approaches may be effective in upper-income communities with relatively low tobacco morbidity and mortality risk, the proposed outreach approach may be especially effective and worth implementing in clinics serving low-income and other high-risk populations that experience greater challenges associated with smoking behavior change.

## Abbreviations

AAR: Ask, advise, refer; AAR + AC: Ask, advise, refer + attention control; AAR + BC: Ask, advise, refer + behavioral counseling; ANCOVA: Analysis of covariance; CEASE: Clinical effort against secondhand smoke exposure; EHR: Electronic health record; FRESH: Family rules for establishing smokefree homes; KiSS: Kids safe and smokefree; PI: Principal Investigator; SHSe: Secondhand smoke exposure.

## Competing interests

The authors declare that they have no competing interests to disclose.

## Authors’ contributions

SJL and BNC developed the study concept and aims. SJL developed the attention control nutrition intervention. BNC developed the behavioral counseling intervention for SHSe reduction and smoking cessation. SJL, BNC, JPW, BM, TB-S, DT, and DF assisted with the operationalization and design of the pediatric clinic intervention, including clinic-specific implementations of the “ask, advise, and refer” protocol and electronic health record modifications. AD assisted with the analytic plan and randomization procedures. SJL drafted the manuscript. SJL, BNC, UN, and MG will oversee and implement the protocol, enrollment, data quality control, data collection, and intervention training and treatment fidelity. All authors contributed to and approved the final manuscript.

## Pre-publication history

The pre-publication history for this paper can be accessed here:

http://www.biomedcentral.com/1471-2458/13/792/prepub
